# Loneliness in the UK during the COVID-19 pandemic: Cross-sectional results from the COVID-19 Psychological Wellbeing Study

**DOI:** 10.1371/journal.pone.0239698

**Published:** 2020-09-24

**Authors:** Jenny M. Groarke, Emma Berry, Lisa Graham-Wisener, Phoebe E. McKenna-Plumley, Emily McGlinchey, Cherie Armour

**Affiliations:** 1 Centre for Improving Health-Related Quality of Life (CIHRQoL), School of Psychology, Queen’s University Belfast, Belfast, United Kingdom; 2 Stress Trauma and Related Conditions (STARC) Research Lab, School of Psychology, Queen’s University Belfast, Belfast, United Kingdom; Fukushima Medical University School of Medicine, JAPAN

## Abstract

**Objectives:**

Loneliness is a significant public health issue. The COVID-19 pandemic has resulted in lockdown measures limiting social contact. The UK public are worried about the impact of these measures on mental health outcomes. Understanding the prevalence and predictors of loneliness at this time is a priority issue for research.

**Method:**

The study employed a cross-sectional online survey design. Baseline data collected between March 23rd and April 24th 2020 from UK adults in the COVID-19 Psychological Wellbeing Study were analysed (N = 1964, 18–87 years, M = 37.11, SD = 12.86, 70% female). Logistic regression analysis examined the influence of sociodemographic, social, health and COVID-19 specific factors on loneliness.

**Results:**

The prevalence of loneliness was 27% (530/1964). Risk factors for loneliness were younger age group (OR: 4.67–5.31), being separated or divorced (OR: 2.29), scores meeting clinical criteria for depression (OR: 1.74), greater emotion regulation difficulties (OR: 1.04), and poor quality sleep due to the COVID-19 crisis (OR: 1.30). Higher levels of social support (OR: 0.92), being married/co-habiting (OR: 0.35) and living with a greater number of adults (OR: 0.87) were protective factors.

**Conclusions:**

Rates of loneliness during the initial phase of lockdown were high. Risk factors were not specific to the COVID-19 crisis. Findings suggest that supportive interventions to reduce loneliness should prioritise younger people and those with mental health symptoms. Improving emotion regulation and sleep quality, and increasing social support may be optimal initial targets to reduce the impact of COVID-19 regulations on mental health outcomes.

## Introduction

On January 31^st^, 2020 the first case of severe acute respiratory syndrome coronavirus 2 (SARS-CoV-2) which causes COVID-19, was confirmed in the UK. On March 23^rd^ a state of lockdown was announced by UK governments across the four devolved nations. Since this time, the UK population has experienced a considerable reduction (and in some cases a complete absence) of in-person social contact. While this acute phase of lockdown will be loosened with decreasing cases of COVID-19, periods of physical distancing are likely to be enforced with new waves of transmission.

With UK mental health services straining to allocate resources to support the growing number of people with mental health problems pre-pandemic; it is predicted that there will be an upsurge of service demand as a result of the psychological sequela of COVID-19 [1]. This is a concern echoed worldwide [2]. In fact, among the UK public, fears surrounding the psychological harms of COVID-19 are ranked above that of physical wellbeing [3]. Prior to the pandemic the UK government had identified loneliness as a significant public health issue, and it has been described as an epidemic [4]. Loneliness is a priority focus if we are to fully understand the psychosocial impact of the COVID-19 pandemic [1].

### Loneliness and COVID-19

As physical distancing rules have resulted in a decline of in-person social contact, it is suggested that rates of loneliness will rise, which may increase prevalence of mood disorders, self-harm, and suicide, and exacerbate pre-existing mental health conditions [1]. Loneliness is associated with worse physical and mental health [5–8] and increases mortality risk [9,10]. While situational loneliness is associated with mortality risk, it is more pronounced in individuals experiencing chronic loneliness [11]. This suggests that, without intervention, prolonged loneliness can have a profound negative impact on health and wellbeing. Systematic review findings recommend that interventions addressing loneliness should focus on individuals who are socially isolated and should target determinants of loneliness which are amenable to change [12].

While existing evidence provides a framework to understand factors which inflate vulnerability to loneliness, we lack a comprehensive understanding of how this might differ in the context of a pandemic. In particular, how psychosocial factors or factors specific to disease-containment policies might elevate or mitigate risk. Moreover, in non-pandemic contexts, evidence suggests that the prevalence of loneliness ranges from 6–76%; with variations across demographic groups [5,13–17] and countries [18,19]. Considering the drastic changes in the current social context, it is conceivable that the prevalence of situational loneliness will be high; which is substantiated by the publics’ concerns regarding the impact of social isolation on mental health [1,3].

### Risk factors for loneliness

Much of what we know in regard to risk factors for loneliness emerges from research with older adults, with a smaller body of research with adolescents and younger adults. Associations between age and loneliness have been positive [20], negative [21], and u-shaped with peaks in younger and older adulthood [19,22]. Findings on gender differences have also been mixed, with some studies reporting higher loneliness in females [23] and others finding no effect of gender [7,8,10]. Risk of loneliness is greater among individuals with mental [24] and chronic physical health conditions [25], however the direction of the effect is unclear.

The COVID-19 crisis presents many challenges for managing feelings of loneliness. Studies of quarantine have found that individuals struggle to adapt to a way of life incongruent with humans’ social nature [26], and report a range of negative psychological reactions to quarantine, including loneliness [27,28]. In non-pandemic contexts, geographical isolation, living alone, and lack of social engagement predicts loneliness in adult and older adult populations [5,13,15,20,29–31]. Limited social interaction is a particularly important risk factor for loneliness among younger people [19,32]. On the other hand, close relationships and social capital have been associated with lower odds of being lonely [7,24]. Redundancy or unemployment due to the pandemic may challenge people’s economic security, and socioeconomic status, lower income, and unemployment have been associated with increased loneliness [7,33]. People working in key roles during the pandemic, especially those in healthcare professions, are under increased pressure, and there is concern that their mental health may be at risk [1]. It is not clear how this might extend to experiences of loneliness. However, one study conducted during the SARS outbreak did find that loneliness was reported by both healthcare workers and non-healthcare workers [34].

Recent evidence in the context of COVID-19 reports high levels of distress and loneliness in US regions with quarantine or shelter-in-place guidelines [35–38]. In the UK, 36% of respondents reported feeling sometimes or often lonely during COVID-19 [39], and Bu et al., [40] found that prevalence of severe loneliness was 14% and remained relatively stable over 6 weeks of lockdown. Being younger, female [39–41], having lower socioeconomic status, a pre-existing mental health condition, and living alone increased the odds of being lonely [40,41]. During physical distancing in Spain, younger people, females, those with less social contact and lower sleep quality reported higher loneliness [21]. Financial concerns and worries about the prolonged impact of quarantine are associated with loneliness; as are feelings of fear, boredom, and uncertainty [1,26,42]. Studies have found that more frequent in-person contact mitigates the impact of the pandemic on loneliness [37,38], and that living with others, larger social network size, and greater social support are protective factors [40]. Furthermore loneliness in the current pandemic context is associated with increased depression, anxiety and suicidal ideation in the US [36,43], and with greater depression, anxiety, and stress in the UK [44]. In Poland, loneliness had a negative impact on mental health symptoms and increased participants’ affective response to aspects of the COVID-19 crisis [42].

### Aims and objectives

Existing evidence surrounding loneliness in the context of COVID-19 has revealed several key determinants of loneliness and the negative impact on mental health outcomes if experiences persist without intervention. There is a need to build on this small body of research. The aim of the current study is to explore the prevalence of loneliness, as well as, risk and protective factors in a UK context. In doing so, this study helps to address key research priorities for the COVID-19 pandemic identified by researchers and the general public [1,3]. This is essential in order to guide an evidence-based public health approach to prevent psychological morbidity as a result of the current and future waves of COVID-19, and may also be an important factor in the public’s ability to adhere to physical distancing regulations over time.

Our primary objectives were to 1) determine the rate of loneliness among adults in the UK during the early stages of the COVID-19 pandemic reported via the COVID-19 Psychological Wellbeing Study and 2) identify differences in sociodemographic, social, health, and COVID-19-specific factors between people with and without loneliness to determine the risk and protective factors for loneliness.

## Methods

### Study design

This study uses data from the COVID-19 Psychological Wellbeing study, an online study of mental health in the UK during the COVID-19 pandemic. The study began on the day the UK lockdown was announced (March 23^rd^) and closed for new entries on April 24^th^ 2020. In the current study, we examine the cross-sectional baseline data of the COVID-19 Psychological Wellbeing study. Full methodological details of the study are reported elsewhere [45]. We used the STROBE cross-sectional checklist when writing this report [46].

### Procedure

The study was approved by the ethical review panel in the faculty of Engineering and Physical Sciences at Queen’s University Belfast (Reference: EPS 20_96) and also Glasgow Caledonian University Health and Life Sciences Ethics Committee, (Reference: HLS/PSWAHS/19/157). Participants were recruited via social media platforms (e.g., Twitter, Facebook). Additional data was collected using a panel of UK residents hosted by Prolific. After providing informed consent participants completed the online survey, which was administered through Qualtrics.

### Participants

There were 2511 responses to the baseline survey. Following screening for inclusion criteria (i.e., UK residents over 18 years of age, informed consent provided) and data quality (i.e., not completing any measures, or having a completion time less than half the median completion time), 522 respondents were removed from the dataset. This resulted in 1989 eligible participants.

Of these eligible participants, 1402 (70.5%) were recruited via Prolific and received compensation for their time (£1–2). Those who were recruited via a social media campaign (29.5%) were included into a prize draw for one of six £150 vouchers. There were some significant, albeit slight, differences in sociodemographic, COVID-19, social and health factors across these two recruitment strategies (S1 Table). Relative to participants recruited through social media, the sample recruited via Prolific had a higher proportion of respondents from England, more males, were younger, had lower self-rated income and education. More of the respondents recruited through Prolific were self-isolating and fewer were in keyworker roles. However, recruitment strategy had no association with level or prevalence of loneliness.

### Measures

#### Loneliness

Loneliness was measured using the Three-Item Loneliness Scale [47]. The scale measures three different aspects of loneliness, (social connectedness, relational connectedness and self-perceived connectedness), with higher scores indicating higher levels of loneliness. Scores above 6 have been used as a cut-off point for loneliness in past research [7,48]. The psychometric properties of the scale are well documented [47,49]. Reliability (i.e., internal consistency) of the measure was high in the current sample (Cronbach’s alpha: α = .83).

#### Sociodemographic variables

Participants provided information on their country of residence, gender, age, self-rated income level (below average, average, above average), employment status (full-time, part-time, unemployed, self-employed [full or part-time], not able to work, retired, student), and highest level of educational attainment (no qualifications, completed secondary school to o-level, GCSE or similar, completed Secondary school to A-level or similar, certificate of Higher Education or similar, Diploma of Higher Education or similar, Undergraduate degree, Postgraduate Degree, Doctoral Degree).

#### COVID-19 variables

Participants were asked to indicate their current living status in relation to COVID-19 at the time of completing the baseline survey (‘I am living as normal’, ‘I am not self-isolating but have cut down my usual activities as a precaution’, ‘I am not self-isolating but have been told to work from home’, ‘I am self-isolating as I do not want to get ill, but I am not high risk, ‘I am self-isolating as I do not want to get ill, but I am regarded as high risk’, ‘I am self-isolating as I do not want others to get ill’, ‘I have been told to self-isolate due to possible symptoms of COVID-19’, ‘I have been told to self-isolate due to a diagnosis of COVID-19’, or ‘I have been ordered by the government or local authority to self-isolate/stay home’). Participants were also asked whether they themselves (at the time of survey completion) are currently in quarantine or have been in the past. Participants were asked if they were caring for someone with COVID-19. Participants were asked if they were working as part of the government assigned key worker roles (including health and social care, education and childcare, transport, public services, government, food, public safety, utilities).

#### Social variables

Levels of social support was measured using the Perceived Social Support Questionnaire-Brief Form [50] The measure contains 6 items which are rated on a 5-point Likert scale, with the response categories ranging from 1 (‘not true at all’) to 5 (‘very true’). Higher scores reflect higher perceived social support. Previous research supports the reliability and validity of the scale [50,51], and reliability was very high (α = .87) in this sample.

Participants were asked about their relationship status (single/never married, married/living with partner, separated or divorced, widowed). Participants were also asked about the type of area they lived in (city, town, rural), and to specify the number of adults over 18 and children under 18 living in their place of residence.

#### Health variables

*Pre-existing physical or mental health conditions*. Participants were asked whether they have ever suffered from several physical or mental health conditions. These included, asthma, heart disease, cancer, diabetes, shortness of breath, several mental health disorders or another kind of chronic condition not specified.

*Post-Traumatic Stress Disorder (PTSD)*. The PTSD Checklist for DSM-5 was used to measure symptoms of PTSD (PCL-5) [52]. The measure contains 20 items, rated on a five-point Likert scale (‘0 = Not at all’ to ‘4 = Extremely’) that mirror the DSM-5 criteria for PTSD. In keeping with previous research [53] a cut off score of 34 was used to indicate ‘probable PTSD’. The excellent psychometric properties of the PCL-5 are well-established [52,54]. Internal consistency of the measure was very high in the current sample (α = .96). To capture post-COVID-19 trauma responses the wording of the PCL-5 was slightly modified (i.e., “Keeping your coronavirus (COVID-19) experiences in mind, please read each problem carefully and indicate how much you have been bothered by that problem IN THE PAST MONTH").

*Generalised anxiety disorder & major depression*. Symptoms of generalised anxiety disorder and major depressive disorder were measured using the seven item Generalised Anxiety Disorder scale (GAD-7) [55] and the nine item Patient Health Questionnaire (PHQ-9) [56]. Previous research has demonstrated the excellent psychometric properties of the GAD-7 [55,57–59] and the PHQ-9 [60–62] across a range of clinical and non-clinical populations. In the current sample reliability was very high for the GAD-7 (α = .94) and for the PHQ-9 (α = .91). Both scales measure symptomatology based on the past two weeks, with item responses ranging from 0 (not at all) to 3 (nearly every day). Across both measures higher scores yield higher degrees of symptom severity, with scores of 10 or more indicating clinical concern [56,63]. This threshold was therefore used in the current study.

*Emotional dysregulation*. The Difficulties in Emotion Regulation Scale—Short Form (DERS-SF) [64] was used to measure emotional dysregulation. The DERS-SF contains 18 items rated on a 5-point likert scale, ranging from 0 to 5. The response categories were, ‘almost never’ (1), ‘sometimes’ (2), ‘about half of the time’ (3), ‘most of the time’ (4), and ‘almost always’ (5). In comparison to the original long form, the psychometric properties of the DERS-SF are excellent [64]. Internal consistency of the measure in the current sample was very high (α = .90).

*Sleep quality*. Sleep quality in general, as well as, sleep quality over the past month in relation to COVID-19 was assessed. Participants were asked to rate their sleep quality in reference to both of these aspects as either ‘very good’, ‘fairly good’, ‘fairly bad’ or ‘very bad’.

### Defining variables

Age was recoded into 6 age range categories (18–24, 25–34, 35–44, 45–54, 55–64, 65+). Employment status was collapsed into those in employment (full-time, part-time, self-employed) versus those who were not currently employed. Educational attainment and self-rated income were treated as continuous variables with higher scores indicating higher attainment and higher income. A new variable was created to identify those participants living alone (versus not living alone). New variables were created for presence of a physical health condition (inclusive of asthma, heart disease, cancer, diabetes and shortness of breath) and mental health condition (inclusive of PTSD, MDD, phobia, social phobia, Obsessive Compulsive Disorder, GAD, psychotic disorder, eating disorder and health anxiety). Research has shown that co-morbidity and multi-morbidity are associated with worse mental health and quality of life [65–67] and greater loneliness [68], as such, new variables were created for the number of physical health and mental health conditions that participants reported. New categorical variables were also created for scores meeting the clinical threshold for depression (scores of 10 or higher on the PHQ-9), anxiety (scores of 10 or higher on the GAD-7) and probable PTSD (scores of 34 or higher on the PCL-5).

### Statistical analysis

Of the 1989 eligible respondents, 1964 completed the measure of loneliness and are the focus of the analyses. As less than 5% of the data were missing pairwise and listwise deletion were implemented [69,70]. In the current study statistical significance was determined as *p* < .05. For interpreting results a more conservative alpha level of .01 may be preferred, given multiple comparisons and in light of the Bonferroni approach. Exact p-values are reported for all tests. When interpreting the findings, the reader should balance the reported significance level with the magnitude of effect, the quality of the study, and with findings of other studies. Absolute numbers, percentages, or means with standard deviations are reported. Unadjusted associations between potential risk factors and loneliness were assessed by the independent *t*-test for continuous variables and the chi-square test for categorical variables. For continuous variables and categorical variables with more than 2 levels, unadjusted odds ratios (ORs) were obtained by separately fitting each variable against the binary loneliness classification (univariate analyses). Factors that were found to be related to loneliness (using a less conservative threshold of *p* ≤ 0.10) were then entered into a multivariable logistic regression model using stepwise backward selection. Multivariable logistic regression examines the contribution of each variable in distinguishing between groups (with or without loneliness), while controlling for the other variables in the model and was used to assess the relative predictive ability of sociodemographic factors, COVID-19 specific factors, social factors, and health factors in explaining prevalence of loneliness.

## Results

### Sample characteristics

Participants were aged 18 to 87 years; average age was 37.11 (SD = 12.86). Participants were mostly white (92.7%) females (70.4%), and not religious (57.5%). All participants were resident in the UK (38.1% were living in England, 36.2% in Scotland, 23.4% in Northern Ireland, and only 2.3% lived in Wales). The majority of respondents were employed (71.9%), however, 37.9% of participants rated their income level as below average. More than half the sample had a university degree (58.5%). Remaining sample characteristics are presented in the first column of Table 1.

**Table 1:** Sample characteristics, and prevalence of loneliness across sociodemographic, COVID-19, social and health factors.

	Sample total	Low/no Loneliness	Loneliness	Unadjusted OR (CI)	ß	*p*
**N (%)**	1964 (100)	1434 (73.4)	530 (26.6)			
**SOCIODEMOGRAPHIC FACTORS**						
**UK nation**						.180*
Northern Ireland	23.4	76.7	23.3	Ref (1.00)		
England	38.1	71.2	28.7	1.33 (1.02, 1.74)	0.29	.035
Scotland	36.2	72.3	27.7	1.27 (0.97, 1.66)	0.24	.088
Wales	2.3	76.1	23.9	1.04 (0.51, 2.11)	0.04	.921
**Gender**						.911*
Male	29.6	73.1	26.9	Ref (1.00)		
Female	70.4	73.4	26.6	1.02 (0.82, 1.26)	0.16	.889
**Age Group**						< .001*
18–24	16.7	59.0	41.0	20.48 (4.92, 85.27)	3.02	< .001
25–34	33.4	71.8	28.2	11.61 (2.81, 48.01)	2.45	.001
35–44	23.9	78.0	22.0	8.30 (1.99, 34.55)	2.12	.004
45–54	14.6	74.8	25.2	9.92 (2.36, 41.65)	2.29	.002
55–64	8.4	79.4	20.6	7.66 (1.78, 32.93)	2.04	.006
65+	3.1	96.7	3.3	Ref (1.00)		
**Employed**	71.9	75.1	24.9	Ref (1.00)		.001*
Not	28.1	67.5	32.5	1.45 (1.17, 1.80)	0.37	.001
**Income**	0.79±0.71	0.86±.72	0.59±.65	0.57 (0.49, 0.66)	-0.56	< .001ˆ
**Educational attainment**	5.22±1.86	5.32±1.84	4.84±1.77	0.87 (0.83, 0.92)	-0.14	< .001ˆ
**COVID-19 FACTORS**						
**Quarantined**	3.7	82.2	17.8	1.73 (0.94, 3.17)	0.55	.081*
Not	96.3	72.8	27.2	Ref (1.00)		
**Self-isolating**	58.9	71.0	29.0	1.28 (1.04, 1.57)	0.25	.020*
Not	41.1	75.8	24.2	Ref (1.00)		
**Self-isolating [High Risk]**						.017*
Yes	9.2	66.1	33.9	1.61 (1.14, 2.28)	0.48	.007
Other reasons	49.7	71.9	28.1	1.22 (0.99, 1.52)	0.20	.062
*Not self-isolating*	*41*.*1*	*75*.*8*	*24*.*2*	Ref (1.00)		
**Self-isolating [by order]**						.049*
Yes	15.4	69.5	30.5	1.37 (1.02, 1.84)	0.32	.033
Other reasons	43.5	71.5	28.5	1.25 (1.00, 1.55)	0.22	.048
*Not self-isolating*	*41*.*1*	*75*.*8*	*24*.*2*	Ref (1.00)		
**Caring [COVID-19]**	5.5	71.3	28.7	1.09 (0.71, 1.68)	0.90	.682*
Not	94.5	73.1	26.9	Ref (1.00)		
**Key worker**	37.4	74.6	25.4	0.88 (0.72, 1.08)	-0.13	.229*
Not	62.6	72.1	27.9	Ref (1.00)		
**SOCIAL FACTORS**						
**Social support**	21.64±5.79	22.75±5.22	18.65±6.19	0.88 (0.87, 0.90)	-0.13	< .001ˆ
**Relationship Status**						< .001*
Single/never married	36.8	59.9	40.1	Ref (1.00)		
Married/co-habiting	56.8	84.1	15.9	0.28 (0.23, 0.35)	-1.27	< .001
Separated/divorced	5.2	53.1	46.9	1.32 (0.87, 2.02)	0.28	.195
Widowed	1.2	65.2	34.8	0.80 (0.33, 1.91)	-0.23	.612
**Household size**						
Number of adults in the home	2.22±0.96	2.25±.92	2.14±1.07	0.89 (0.80, 0.98)	-0.12	.026ˆ
Number of children in the home	1.63±0.95	1.66±.98	1.54±0.85	0.87 (0.78, 0.97)	-0.14	.011ˆ
**Living alone**	14.8	57.9	42.1	2.25 (1.74, 2.92)	0.81	< .001*
Not	85.2	75.6	24.4	Ref (1.00)		
**Urbanicity**						.375*
Rural	21.9	71.1	28.9	Ref (1.00)		
Town	44.0	74.5	25.5	0.84 (0.65, 1.09)	-0.17	.192
City	34.2	72.3	27.8	0.94 (0.72, 1.23)	-0.06	.670
**HEALTH FACTORS**						
**Physical Health Condition**	24.8	69.3	30.7	1.27 (1.01, 1.59)	0.24	.036*
None	75.2	74.2	25.8	Ref (1.00)		
**Number of health conditions**	0.29±0.55	0.28±0.55	0.33±.058	1.18 (1.00, 1.41)	0.17	.057ˆ
**Mental Health condition**	30.6	59.0	41.0	2.64 (2.14, 3.25)	0.97	< .001*
None	69.4	79.2	20.8	Ref (1.00)		
**Number of mental health conditions**	0.55±1.05	0.42±0.91	0.92±1.31	1.50 (1.37, 1.64)	0.41	< .001ˆ
**Depression—clinical threshold**	34.0	49.2	50.8	5.98 (4.82, 7.42)	1.79	< .001*
Not	66.0	85.3	14.7	Ref (1.00)		
**Anxiety–clinical threshold**	30.3	52.2	47.8	4.18 (3.38, 5.17)	1.43	< .001*
Not	69.7	82.0	18.0	Ref (1.00)		
**Probable PTSD**	19.4	43.8	56.2	5.13 (4.05, 6.51)	1.63	< .001*
Not	80.6	80.0	20.0	Ref (1.00)		
**Emotion regulation difficulties**	42.43±13.22	39.17±11.67	51.20±13.14	1.08 (1.07, 1.09)	0.74	< .001ˆ
**Sleep quality [general]**	2.22±0.79	2.10±.75	2.54±.81	2.03 (1.78, 2.32)	0.71	< .001ˆ
**Sleep quality [COVID-19]**	2.47±0.84	2.34±.80	2.84±.81	2.13 (1.87, 2.43)	0.76	< .001ˆ

Notes

* = X^2^ test

ˆ = independent samples *t*-test. Numerical values with standard deviation are mean scores, values without standard deviation are percentages. OR = unadjusted odds ratio. CI = 95% confidence intervals. ß = regression coefficient.

### Loneliness prevalence

**Fig 1:**
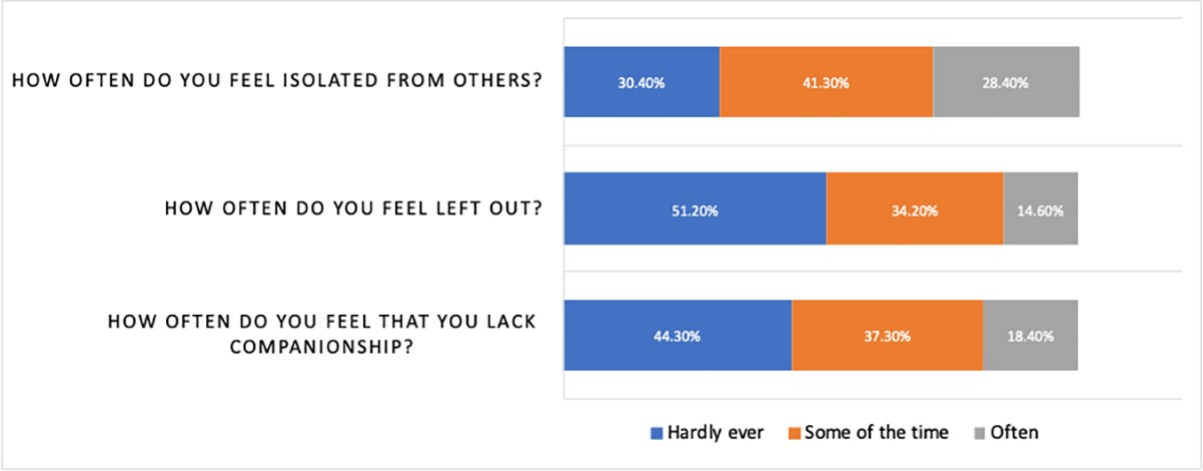
Percentage of responses to each item of the 3-item UCLA loneliness scale.

Loneliness was defined as having a score on the 3-item loneliness scale in the top quartile (i.e., a score of 7 or higher). The overall prevalence of loneliness was 27% (530/1964). The mean score was 5.36 (SD = 1.92). In the past week 49% to 70% of respondents reported feeling isolated, left out, or lacking companionship some of the time or often (Fig 1).

### Risk factors for loneliness

#### Univariate analyses

**Fig 2:**
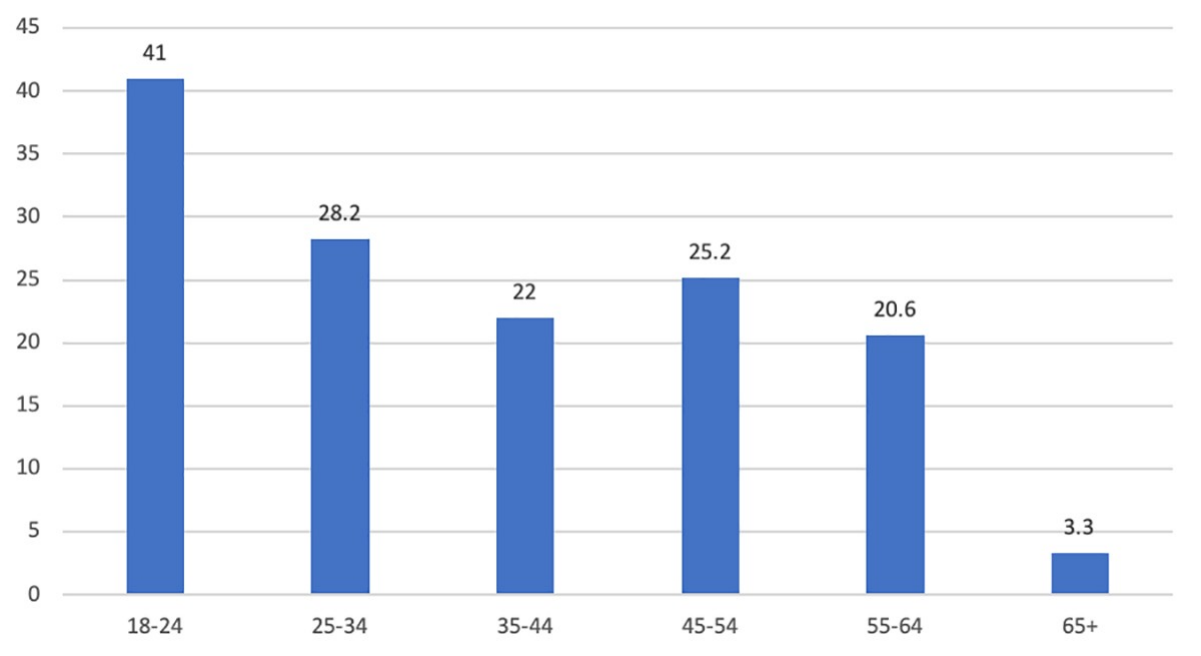
Prevalence of loneliness (%) by age group.

Table 1 compares participants with (26.6%) and without loneliness (73.4%) on sociodemographic factors, factors specific to COVID-19, social factors and health factors. Overall country of residence had no impact on loneliness, however, relative to living in Northern Ireland living in England increased the odds of being lonely (OR: 1.33, CI: 1.02, 1.74). The prevalence of loneliness decreased with age (Fig 2), with 18-24-year old’s having the highest frequency of loneliness (41%), whereas only 3% of people over 65 were classified as lonely. There was no association between gender and loneliness. Loneliness was associated with lower income, lower educational attainment and was more prevalent in people out of employment. In relation to COVID-19, prevalence of loneliness was higher for those self-isolating, and for those self-isolating because they are considered high risk or have been ordered to self-isolate. However, loneliness was not related to being in or having been in quarantine. Further, odds of loneliness were not higher for key workers, or for people caring for someone with COVID-19. Loneliness was less frequent in people who are married or living with a partner. There was an inverse relationship between household size and loneliness. Living alone more than doubled the odds of being lonely. People who were lonely also had lower perceived social support. Odds of loneliness were higher for those with pre-existing physical and mental health conditions. Rates of loneliness were twice as high among people whose scores met clinical criteria for depression, anxiety and probable PTSD. Higher emotion regulation difficulties and lower sleep quality were also associated with loneliness.

#### Multivariable analysis

The 23 factors that were significant at the alpha level ≤ .10 in the univariate analyses were entered into multivariable logistic regression. The final model with adjusted odds ratios (ORs) with 95% confidence intervals (CIs) for the various predictors is shown in Table 2.

**Table 2:** Multivariable logistic regression analysis of factors associated with loneliness.

	ß	aOR (CI)	*p*
**Constant**	-2.73	0.06	.002
**Age Group**			
18–24	**1.67**	5.31 (1.13, 24.96)*	**.034**
25–34	**1.54**	4.67 (1.02, 21.33)*	**.047**
35–44	1.31	3.71 (0.81, 17.00)	.091
45–54	**1.56**	4.75 (1.03, 21.92)*	**.046**
55–64	1.13	3.10 (0.65, 14.81)	.156
65+		Ref (1.00)	
**Social support**	**-0.08**	0.92 (0.90, 0.94)**	**< .001**
**Relationship Status**			
Single/never married		Ref (1.00)	
Married/co-habiting	**-1.06**	0.35 (0.26, 0.46)**	**< .001**
Separated/divorced	**0.83**	2.29 (1.31, 4.00)*	**.004**
Widowed	0.69	1.99 (0.69, 5.72)	.200
**Household size**			
Number of adults living in the home	**-1.38**	0.87 (0.76, 1.00)**	**.046**
**High depression**	**0.55**	1.74 (1.24, 2.44)*	**.001**
**High anxiety**	0.31	1.34 (0.96, 1.92)	.085
**Emotion regulation difficulties**	**0.04**	1.04 (1.03, 1.05)*	**< .001**
**Sleep quality [COVID]**	**0.26**	1.30 (1.09, 1.55)*	**.003**
**Probable PTSD**	0.33	1.39 (0.97, 1.98)	.071

*Notes;* aOR = adjusted odds ratio; CI = confidence intervals; ß = regression coefficient

* = risk factor and

** = protective factor.

Relative to those over 65 years of age, younger adults were 4–5 times more likely to be lonely (18–24 [OR]: 5.31, CI: 1.13–24.96], 25–34 [OR: 4.67, CI: 1.02–21.33], or 45–54 [OR: 4.75, CI: 1.03–21.92]). Compared to being single, being separated or divorced more than doubled the odds of being lonely (OR: 2.29, CI: 1.31–4.00), whereas being married or cohabiting was associated with lower odds of being lonely (OR: 0.35, CI: 0.26–0.46). Odds of loneliness decreased with greater number of adults living in the same home (OR: 0.87, CI: 0.76–1.00) and with higher levels of perceived social support (OR: 0.92, CI: 0.90–0.94). People meeting clinical criteria for diagnosis of major depressive disorder were almost twice as likely to be lonely (OR: 1.74, CI: 1.24–2.44). Greater difficulties with emotion regulation (OR: 1.04 CI:1.03–1.05), and worse quality sleep due to the COVID-19 situation (OR: 1.30 CI: 1.09–1.55) also increased the odds of being lonely.

## Discussion

The overall prevalence of loneliness in this sample was 27%. In univariate analyses, younger age, lower income, unemployment, less education, relationship status, smaller household size, living alone, lower social support, having a physical or mental health condition, meeting clinical criteria for depression, anxiety and PTSD, emotion regulation difficulties and poor sleep quality were all associated with loneliness. Self-isolating for any reason, including being high-risk or having been advised to shield was also associated with loneliness. In the adjusted analyses while controlling for all other factors, younger age group, being separated/divorced, scores indicative of depression, poor sleep quality due to COVID-19 and difficulties in emotion regulation were significant risk factors for loneliness. Whereas, being married or co-habiting, living with a greater number of adults, and having higher levels of perceived social support were protective factors.

Research prior to the pandemic estimated the prevalence of loneliness to be between 6 and 76%. The rate of loneliness reported here is the same as that found by Victor and Yang [19] in their pre-pandemic research with 2393 adults in the UK (aged 15–97 years). Using a single-item measure of loneliness they found that prevalence of loneliness was 27%. Considering research during the pandemic, prevalence in the current study falls between rates in the US (43%) and the UK (14%-36%) [36,39,40]. There is emerging evidence from the US suggesting that levels of loneliness are high but stable during the COVID-19 pandemic [71].

### Sociodemographic factors

Studies of loneliness during COVID-19 report higher prevalence among females [21,39,40]. Outside of the current crisis, findings on gender differences in loneliness are mixed, and findings of this study conform with those showing no association [7,8,48]. As expected, lower income and less education were associated with loneliness. Adjusting for other significant predictors, these socioeconomic factors were not significant risk factors for loneliness. A recent study by Shovestul et al. [72] found that relative to other demographic and socioeconomic factors, age is the most important risk factor for loneliness. Similarly, in the multivariable analyses, regression coefficients show that age group was the strongest predictor of loneliness in this study. Despite research showing that the relationship between age and loneliness is u-shaped [19,22], we found an inverse relationship between age and loneliness, with very high prevalence of loneliness in the younger age groups and very low rates of loneliness among the over-65s. This is in keeping with studies of COVID-19, showing higher loneliness in younger people [21,40,41]. Younger adults may be disproportionately affected by disease-containment policies (e.g., school/university closures) that increase social isolation placing them at higher risk of loneliness [32]. However, as older adults and males were underrepresented in this study sample, we cannot offer definitive conclusions as to age or gender differences in the impact of lockdown [45].

### Social factors

All the protective factors for loneliness were social variables, supporting a link between social isolation and loneliness. This is in keeping with recent research on loneliness in the UK during the pandemic, that also found social factors were protective [39,40]. The particular variables that were significant predictors in this study (i.e., being married/co-habiting, number of adults living in the household, availability of social support) indicate that the closeness and quality of relationships may be important. It may also indicate that face-to-face interactions are key. In a study of the impact of COVID-19 restrictions in the US, frequent in-person interactions were associated with lower loneliness, but not remote or virtual interactions [37]. It will be difficult to develop interventions to reduce loneliness targeting these social factors, at least until physical distancing regulations are relaxed.

### Health factors

There are numerous cross-sectional studies showing that loneliness is more prevalent among people with mental health conditions [73,74]. There is also compelling evidence that loneliness precedes depression [6]. Many explanations are put forward for this link, for example, the stigma of loneliness may cause those who are already marginalised due to their mental illness to withdraw further, or perhaps behavioural symptoms of depression make social participation more burdensome [75]. During the COVID-19 pandemic loneliness is a significant risk factor for depression, anxiety, stress, mental health symptoms and suicidal ideation [36,42–44]. In the current study of adults in the UK during the COVID-19 crisis, meeting the clinical threshold for major depressive disorder was a significant risk factor for loneliness. Having difficulty regulating emotions also significantly increased the odds of being lonely, and is worthy of further investigation as a potential mechanism of the relationship between isolation, loneliness, and mental health. Longitudinal studies will be necessary to disentangle the temporal dynamics linking loneliness and mental health outcomes throughout different phases of the government lockdown.

### COVID-19 specific factors

Being a keyworker during the pandemic has been associated with worse mental health outcomes [45,76,77]. However, in spite of additional stressors, key workers were at no greater risk for loneliness in this study. There is a link between social isolation and loneliness [32], and quarantine has been associated with negative psychological effects [26–28]. Therefore, it was unexpected that in this study quarantine was not associated with loneliness, and in the adjusted analyses self-isolating was no longer a significant risk factor for loneliness. It is possible that the negative impact of quarantine and isolation on loneliness and mental health may be more pronounced in children and adolescents [78]. Another possible explanation is that the COVID-19 pandemic is unique in the sense that unlike previous outbreaks the disease-containment policies were applied at a population level, irrespective of disease status. The universal nature of the UK lockdown may have mitigated its impact on loneliness. Poor quality sleep due to COVID-19, however, remained a significant risk for loneliness. This is in line with studies in Greece and France that have reported sleep problems are common during this pandemic, and that loneliness is a major contributor to insomnia [79,80]. Indeed, sleep problems have recently been proposed as a mechanism of the relationship between loneliness and health [81].

Overall, factors specific to COVID-19 were not significant predictors of loneliness. This is consistent with a recent study showing that the same risk factors predicted loneliness before and during the pandemic [41]. Together this suggests that interventions to reduce the negative impact of the lockdown should target those who are most at-risk of loneliness outside of the current crisis–that being the young, unemployed, people with low income or education, and people with mental health conditions (i.e., depression). These findings also suggest that existing interventions to reduce loneliness may be effective in this context also [12,82]. Of the risk factors identified in the current study, difficulties in emotion regulation and sleep quality may be the most appropriate to target as they are amenable to change, for example, through cognitive behavioural interventions [83,84].

### Strengths

This study is timely and contributes to a small body of emerging research evidencing prevalence and determinants of loneliness during the pandemic in the UK [39,40], thereby addressing key research priorities for understanding the mental health impact of the pandemic identified by the UK public and the academic community [1,3]. The study identifies a number of significant risk and protective factors for targeted intervention and provides support for existing research in this area through use of a large sample and confirmatory analysis using a well-validated measure of loneliness [47].

### Limitations

1. The sample was not randomly selected. This is typical of existing research in the area (e.g., [40,41], and reflects a rapid emergency data collection exercise [45]. When comparisons were made to the UK census data, older adults and males were found to be under-represented in the sample, and this may be of particular importance in the context of loneliness research.
2. This study focused on experiences of loneliness ‘in the past week’. Due to the COVID-19 restrictions on social contact some people will be experiencing severe and sustained loneliness for the first time. Future studies using alternative designs will be needed to distinguish people experiencing loneliness because of COVID-19 from those who are chronically lonely.
3. Due to the survey being conducted online there was a reliance on self-reported measures of diagnoses and mental health symptoms. That being said, the measures selected have well-established psychometric properties. Related to this limitation is the issue that with much research moving online in response to physical distancing regulations, prospective participants who do not have access to computers and the internet are excluded. Without opportunities for digital alternatives for social contact these people may be particularly isolated and at risk for loneliness during the COVID-19 lockdown. It is important to consider the impact of the digital divide on study findings on the impact of the lockdown.
4. The cross-sectional design of the study means we cannot determine causality. This is particularly pertinent with regard to the ongoing debate as to whether loneliness causes mental health conditions, is a mental health condition in itself, or results from mental health symptoms [82]. It will be important to understand this within the context of COVID-19 also.

## Conclusion

The UK public are concerned about the impact of the lockdown on their mental health [1,3]. More than one quarter of the respondents in the COVID-19 Psychological Wellbeing Study were classified as lonely, suggesting that UK lockdown policies have had a negative impact. In the absence of longitudinal studies examining the same cohort before and after the lockdown this interpretation remains speculative. Being younger, separated or divorced, meeting the clinical threshold for major depressive disorder, having poor quality sleep and difficulties regulating emotions were significant risk factors for loneliness during the initial stage of the lockdown. However, being married, living with a partner or other adults, and having greater social support were protective. Our findings suggest that supports aimed at improving emotion regulation, sleep quality, and increasing social support may be the most impactful for mitigating the mental health impact of the lockdown, and that interventions should focus on those people most at-risk for loneliness prior to the lockdown.

## Supporting information

S1 Table Prevalence of loneliness and sample characteristics across recruitment strategy. (DOCX)
