# Supplementary material for: Loneliness in the UK during the COVID-19 pandemic: Cross-sectional results from the COVID-19 Psychological Wellbeing Study
Source: PLoS One. 2020 Sep 24;15(9):e0239698. doi: 10.1371/journal.pone.0239698 (PMC7513993; doi:10.1371/journal.pone.0239698)
Supplement: S1 Table — (DOCX) [file pone.0239698.s001.docx]

| **Supplementary Table 1.**  Prevalence of loneliness and sample characteristics across recruitment strategy. | | | |
| --- | --- | --- | --- |
|  | Prolific | Social Media | *p* |
| **N (%)** | 1402 (70.5) | 587 (29.5) |  |
|  |  |  |  |
| **Loneliness** | 27.4 | 26.0 | .538* |
| Not | 72.6 | 74.0 |  |
| **Loneliness** | 5.39±1.92 | 5.27±1.93 | .216^ |
| **SOCIODEMOGRAPHIC FACTORS** |  |  |  |
| **UK nation** |  |  | <.001* |
| Northern Ireland | 16.5 | 40.5 |  |
| England | 51.5 | 4.3 |  |
| Scotland | 28.9 | 54.7 |  |
| Wales | 3.1 | 0.5 |  |
| **Gender** |  |  | <.001* |
| Male | 35.0 | 16.4 |  |
| Female | 65.0 | 83.6 |  |
| **Age Group** |  |  | <.001* |
| 18-24 | 18.7 | 11.8 |  |
| 25-34 | 34.6 | 29.6 |  |
| 35-44 | 23.6 | 24.7 |  |
| 45-54 | 13.9 | 16.4 |  |
| 55-64 | 7.2 | 11.6 |  |
| 65+ | 2.0 | 6.0 |  |
| **Employed** | 29.1 | 26.2 | .209* |
| Not | 70.9 | 73.8 |  |
| **Income** | 0.72±0.64 | 0.98±0.76 | <.001ˆ |
| **Educational attainment** | 4.86±1.80 | 5.96±1.70 | <.001ˆ |
| **COVID-19 FACTORS** |  |  |  |
| **Quarantined** | 3.3 | 4.9 | .117* |
| Not | 96.7 | 95.1 |  |
| **Self-isolating** | 60.7 | 53.6 | .004* |
| Not | 39.3 | 46.4 |  |
| **Self-isolating**  **[High Risk]** |  |  | .011* |
| Yes | 9.2 | 9.1 |  |
| Other reasons | 51.5 | 44.5 |  |
| *Not self-isolating* | *39.3* | *46.4* |  |
| **Self-isolating**  **[by order]** |  |  | .014* |
| Yes | 15.9 | 13.9 |  |
| Other reasons | 44.8 | 39.7 |  |
| *Not self-isolating* | *39.3* | *46.4* |  |
| **Caring [COVID-19]** | 5.6 | 5.5 | .904* |
| Not | 94.4 | 94.5 |  |
| **Key worker** | 35.2 | 42.8 | .001* |
| Not | 64.8 | 57.2 |  |
| **SOCIAL FACTORS** |  |  |  |
| **Social support** | 21.17±5.72 | 22.83±5.81 | <.001ˆ |
| **Relationship Status** |  |  | <.001* |
| Single/never married | 39.0 | 31.0 |  |
| Married/co-habiting | 55.9 | 58.9 |  |
| Separated/divorced | 4.2 | 7.9 |  |
| Widowed | 0.9 | 2.2 |  |
| **Household size** |  |  |  |
| Number of adults in the home | 2.27±0.98 | 2.08±0.90 | <.001ˆ |
| Number of children in the home | 1.67±0.96 | 1.52±0.91 | .001ˆ |
| **Living alone** | 13.2 | 19.2 | .001* |
| Not | 86.8 | 80.8 |  |
| **Urbanicity** |  |  | <.001* |
| Rural | 21.0 | 24.1 |  |
| Town | 46.9 | 36.1 |  |
| City | 32.2 | 39.9 |  |
| **HEALTH FACTORS** |  |  |  |
| **Physical Health Condition** | 25.0 | 24.0 | .632* |
| None | 75.0 | 76.0 |  |
| **Number of health conditions** | 0.29±0.55 | 0.29±0.55 | .835ˆ |
| **Mental Health condition** | 29.3 | 33.4 | .072* |
| None | 70.7 | 66.6 |  |
| **Number of mental health conditions** | 0.54±1.05 | 0.59±1.03 | .301ˆ |
| **Depression - clinical threshold** | 34.4 | 33.0 | .570* |
| Not | 65.6 | 67.0 |  |
| **Anxiety – clinical threshold** | 29.2 | 32.7 | .125* |
| Not | 70.8 | 67.3 |  |
| **Probable PTSD** | 18.4 | 22.0 | .070* |
| Not | 81.6 | 78.0 |  |
| **Emotion regulation difficulties** | 42.99±13.26 | 40.99±12.99 | .003ˆ |
| **Sleep quality [general]** | 2.24±0.79 | 2.16±0.77 | .028ˆ |
| **Sleep quality**  **[COVID-19]** | 2.48±0.83 | 2.46±0.86 | .700ˆ |

*Notes;* * = X^2^ test; ˆ = independent samples *t*-test. Numerical values with standard deviation are mean scores, values without standard deviation are percentages.
